# High glucose enhances the metastatic potential of tongue squamous cell carcinoma via the PKM2 pathway

**DOI:** 10.18632/oncotarget.22907

**Published:** 2017-12-04

**Authors:** Wei Wang, Qianting He, Wangxiang Yan, Jingjing Sun, Zujian Chen, Zhonghua Liu, Zhiyuan Lu, Jinsong Hou, Yisen Shao, Xiaofeng Zhou, Anxun Wang

**Affiliations:** ^1^ Department of Oral and Maxillofacial Surgery, First Affiliated Hospital, Sun Yat-Sen University, Guangzhou, China; ^2^ Center for Molecular Biology of Oral Diseases, College of Dentistry, University of Illinois at Chicago, Chicago, IL, USA; ^3^ Department of Oral and Maxillofacial Surgery, Guanghua School of Stomatology, Hospital of Stomatology, Sun Yat-Sen University, Guangzhou, China; ^4^ Department of Oral and Maxillofacial Surgery, Affiliated Hospital of Jiangxi University of Traditional Chinese Medicine, Nanchang, China

**Keywords:** tongue squamous cell carcinoma, metastasis, high glucose, PKM2

## Abstract

Previous evidence has indicated an increased cancer risk in individuals with diabetes mellitus (DM). The aim of this study was to investigate the relationship between DM (high glucose) and tongue squamous cell carcinoma (TSCC) and how high glucose mediated the metastatic potential of TSCC. The relationship between DM and TSCC was assessed in a retrospective study. The role and its mechanism of high glucose on the proliferation, metastatic potential of TSCC were investigated *in vitro* and *in vivo*. The prevalence rate of DM in patients with TSCC was 12.84%, which was significantly higher than that (9.7%) in the general population in China. Although no significant difference was observed in the overall survival (OS) rate, TSCC patients with DM have a 1.38-fold increase in relative risk affecting 5-year OS compared to patients without DM. High glucose enhanced the TSCC cell proliferation, migration, invasion and upregulated PKM2 (pyruvate kinase M2) expression. Whereas, these effect was abolished after knockdown of PKM2 in TSCC cells. High glucose promoted tumour growth and lung metastasis of TSCC in a DM animal model. Our results confirm DM as a risk factor for the development of TSCC. High glucose enhances the metastatic potential of TSCC through stimulation of the PKM2 pathway.

## INTRODUCTION

Diabetes mellitus (DM) is one of the most common chronic diseases in nearly all countries. In 2010, the world prevalence rate of diabetes among adults (aged 20-79 years) was 6.4% [1]. A national study among Chinese adults revealed that the age- and sex-standardized prevalence of total diabetes and prediabetes (i.e., impaired fasting glucose or impaired glucose tolerance) was 9.7% and 15.5%, respectively [2]. These findings indicate the importance of diabetes as a public health problem. Recent epidemiological evidence suggests that DM may contribute to the initiation and propagation of certain cancers [3]. Indeed, individuals with diabetes have a significantly higher likelihood of developing a range of cancers including liver, pancreatic, colorectal and breast cancer [4–7]. Epidemiological studies have also implicated DM as a risk factor for the development of oral squamous cell carcinoma (OSCC) [8], but whether DM affects the progression and prognosis of tongue squamous cell carcinoma (TSCC) remains unclear.

DM is a pathophysiological state of oxidative stress and DNA damage that can lead to various types of mutations, which then cause aberrations in cells and an increased cancer risk [9]. However, the underlying cellular and molecular mechanisms of DM-mediated tumour development remain unclear. Hyperglycaemia (High glucose), a characteristic feature of DM, has been shown to contribute to an enhanced risk of cancer [10]. They found that hyperglycaemia in cancer patients contributes to an increased likelihood of tumour recurrence and metastasis [10]. Many endeavour had been done to uncover the mechanism of how hyperglycaemia mediated proliferation and metastatic in cancer [11, 12]. Recent research found that hyperglycaemia induces the expression of the glycolytic enzyme HK2, which enhances cancer metastasis [13]. PKM2 (pyruvate kinase 2), another key glycolytic enzyme, had been revealed to play an important role in cancer metabolism, tumour growth, invasion and metastasis [14, 15]. In our previous studies, we had showed that PKM2 deregulation plays an important role in patients with TSCC. Overexpression of PKM2 is associated with cervical lymph node metastasis and an unfavourable prognosis in patients with TSCC. PKM2 enhances the metastatic potential of TSCC through the SOD2-H_2_O_2_ pathway [16].

To further investigate the relationship between DM (high glucose) and TSCC and its mechanism of how high glucose mediates TSCC metastasis. We analyzed the relationship between DM and TSCC in a retrospective study. Then, we investigated the role of high glucose in the metastatic potential of TSCC *in vitro* and *in vivo*. Finally, we analyzed the mechanism of how high glucose mediates metastasis in TSCC. We found a high prevalence rate of DM in patients with TSCC. High glucose enhances the metastatic potential of TSCC through stimulation of the PKM2 pathway.

## RESULTS

### High prevalence rate of DM in patients with TSCC

In all, 501 patients with TSCC were included in this retrospective study. As shown in Table 1, the prevalence rate of DM was 13.97%, and the prevalence rate of IFG (Impaired Fasting Glucose) was 20.76%. The prevalence rate according to the age- and sex-adjusted standardized prevalence rate (SPR) of DM in patients with TSCC was 12.84%, and the SPR of IFG was 18.88%. The SPRs of both DM and IFG in TSCC were significantly higher (*P*<0.001) than those in the general Chinese population, in which the SPRs were 9.7% and 15.5% for DM and IFG, respectively. Except for gender, no significant difference was found in regards to age, tumour size, lymph node metastasis, clinical stage or histological grade between TSCC patients with and without DM (Supplementary Table 1). Although no significant difference was observed in the overall survival (OS) rate between DM and non-DM patients with TSCC, the non-DM group showed a better survival trend than the DM group (Data not shown). Moreover, Cox regression revealed that TSCC patients with DM have a 1.38-fold increase in relative risk affecting the 5-year OS compared to patients without DM (Table 2).

**Table 1:** The prevalence rate of DM and IFG in patients with TSCC

	Prevalence rate		SPRs^*^		SPRs in the Chinese population^#^	
	DM	IFG	DM	IFG	DM	IFG
Total	13.97%	20.76%	12.84%	18.88%	9.7%	15.5%
Male (%)	11.19%	17.13%	11.98%	18.34%	10.6%	16.1%
Female (%)	17.69%	25.58%	13.67%	19.40%	8.8%	14.9%

^*^SPRs: standardized prevalence rates. ^#^: Data from Ref [2].

**Table 2:** Results of the univariate and multivariate analyses of factors affecting the 5-year overall survival of patients with TSCC

Characteristics	Univariate analyses	Multivariate analyses		
	*P* value	*P* value	HR	*95%CI*
Gender (Female vs Male)	0.590	0.174	1.29	(0.935, 2.685)
Age (>40 vs ≤40)	0.112	0.087	1.59	(0.894, 1.860)
Tumour size (T_1+2_ vs T_3+4_)	<0.001	0.017	2.01	(1.136, 3.570)
Lymph node metastasis (Positive vs Negative)	<0.001	0.002	2.18	(1.329, 3.589)
Clinical stage (C_I+II_ vs C_III+IV_)	<0.001	0.571	0.83	(0.425, 1.602)
Histological grade (Moderate/Poor vs Well)	<0.001	0.001	1.80	(1.259, 2.579)
DM (Positive vs Negative)	0.090	0.170	1.38	(0.870, 2.199)

Moreover, we investigate the correlation between PKM2 expression and DM in patients with TSCC. The data of PKM2 expression in TSCC detected by IHC had been shown in our previous study [16]. As shown in Supplementary Table 2, strong correlations were found between PKM2 expression and DM in patients with TSCC. We also investigate the correlation between DM and lymph node metastasis in patients with TSCC. As shown in Supplementary Table 3, no correlations were found between DM and lymph node metastasis in patients with TSCC.

### High glucose promotes the proliferation, migration and invasion of TSCC

**Figure 1:**
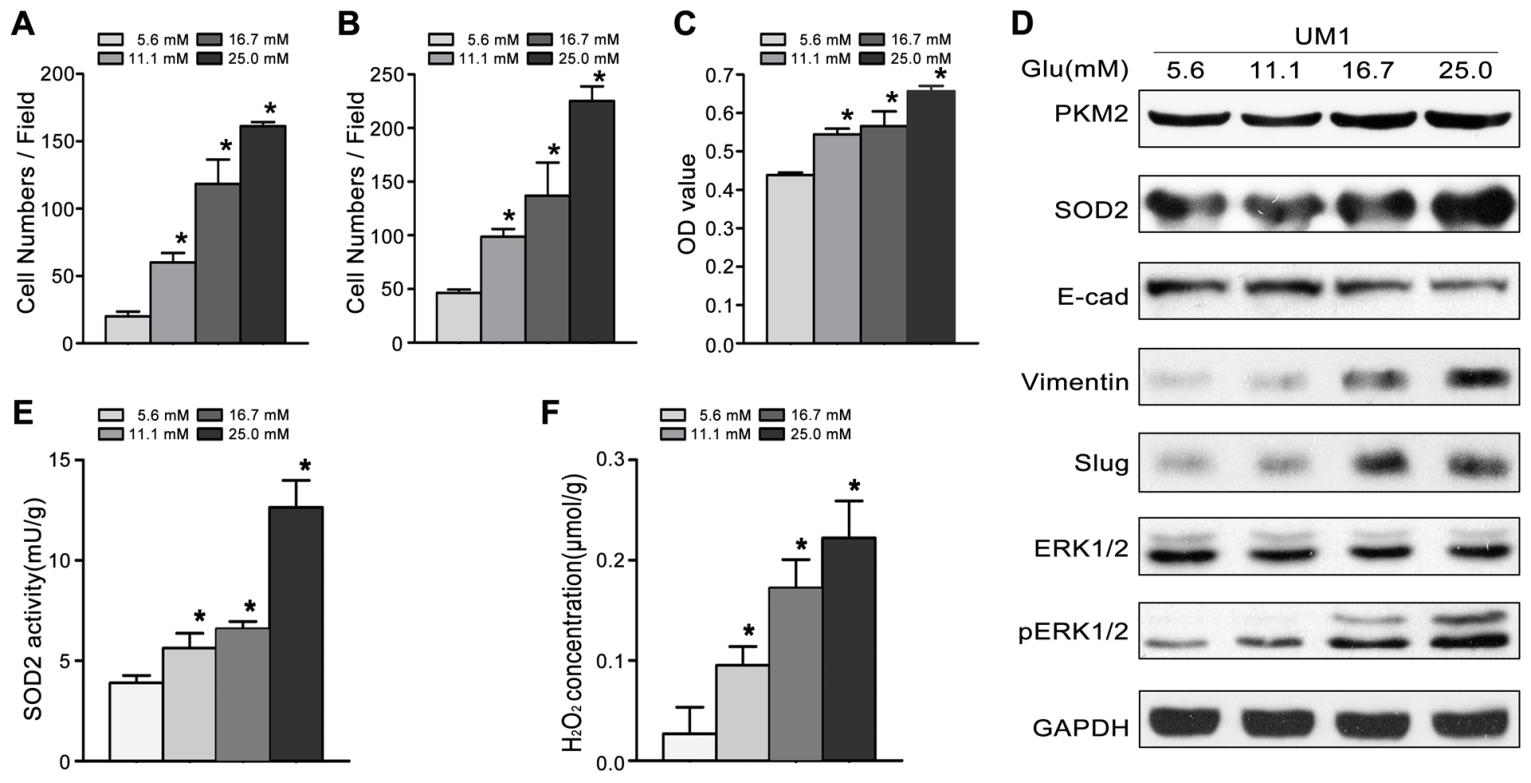
High glucose promotes the migration and invasiveness of TSCC *in vitro* **(A-C)** Compared with normal glucose (5.6mM), high glucose (11.1mM, 16.7mM or 25.0mM) significantly promoted the migration (A), invasion (B) and proliferation (C) of UM1 cells in a concentration - dependent manner. **(D)** The protein levels of PKM2, SOD2, Vimentin, Slug and pERK1/2 were obvious increased in UM1 cells cultured with high glucose (11.1mM, 16.7mM or 25.0mM), but the protein level of E-cadherin was decreased. SOD2 activity **(E)** and H_2_O_2_ production **(F)** were significantly increased in UM1 cells cultured with high glucose (11.1mM, 16.7mM or 25.0mM). ^*^P<0.05 compared with UM1 cells cultured with 5.6mM glucose.

To investigate the role of high glucose in the migration/invasiveness of TSCC, we cultured UM1 cells with different glucose levels (5.6mM, 11.1mM, 16.7mM or 25.0mM). As shown in Figure 1, high glucose significantly increased the migration (Figure 1A) and invasion (Figure 1B) abilities, and proliferation capacity (Figure 1C) in a concentration - dependent manner. High glucose also increased the expression of PKM2 and SOD2 (Figure 1D), the activity of SOD2 (Figure 1E) and production of H_2_O_2_ (Figure 1F) in UM1 cells. Moreover, the expression levels of metastasis-related proteins (pERK1/2, Slug and Vimentin) were clearly increased, while the E-cadherin protein levels were obviously decreased after UM1 cells were cultured with higher glucose (Figure 1D).

### High glucose promotes the proliferation, migration and invasion of TSCC through the PKM2 Pathway

**Figure 2:**
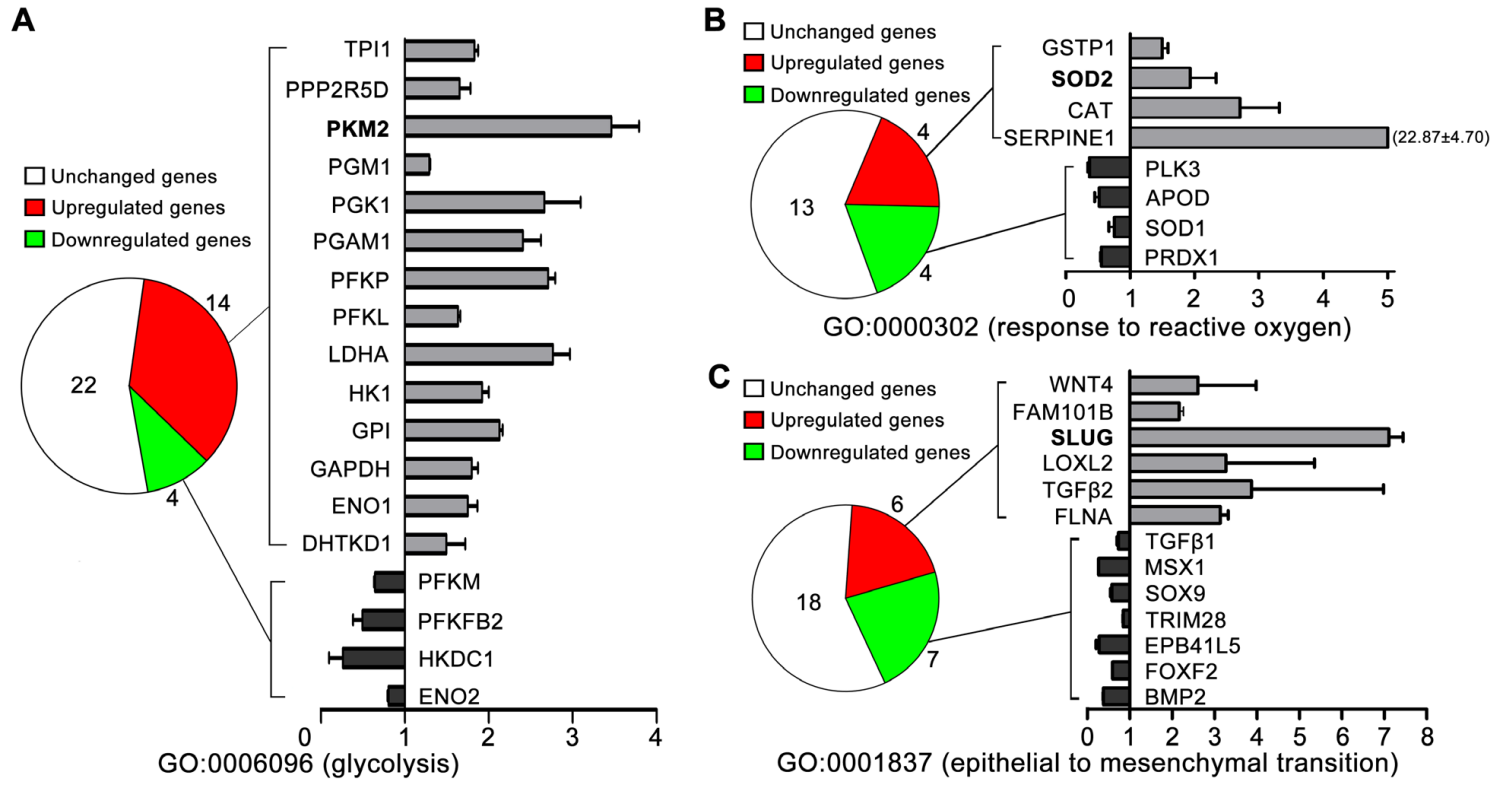
The gene expression patterns of TSCC cells cultured with normal glucose (5.6mM) and high glucose (16.7mM) determined by microarray analysis **(A)** 14/40 of the glycolytic related gene (GO0006096, glycolysis) were upregulated in TSCC cells cultured in 16.7mM glucose compared with those TSCC cells cultured in 5.6mM glucose, including PKM2. The expression of SOD2 [GO0000302, response to reactive oxygen species **(B)**], Slug [GO0001837, epithelial to mesenchymal transition **(C)**] were also found to be upregulated in 16.7mM glucose cultured TSCC cells than in 5.6mM glucose cultured TSCC cells.

To further investigate whether high glucose mediated the metastatic potential of TSCC though PKM2 (glycolytic enzyme) pathway, we detected gene expression patterns by microarray. As shown in Figure 2A, gene ontology (GO) analysis revealed that 14/40 of the glycolytic related gene (GO0006096, glycolysis) were upregulated in TSCC cells cultured in 16.7mM glucose (high glucose) compared with those cultured in 5.6mM glucose (normal glucose), including PKM2, HK1 and LDHA. The expression of SOD2 (GO0000302, response to reactive oxygen species, Figure 2B), Slug (GO0001837, epithelial to mesenchymal transition, Figure 2C) were also found to be upregulated in high glucose (16.7mM) cultured TSCC cells than in normal glucose (5.6mM) cultured TSCC cells.

**Figure 3:**
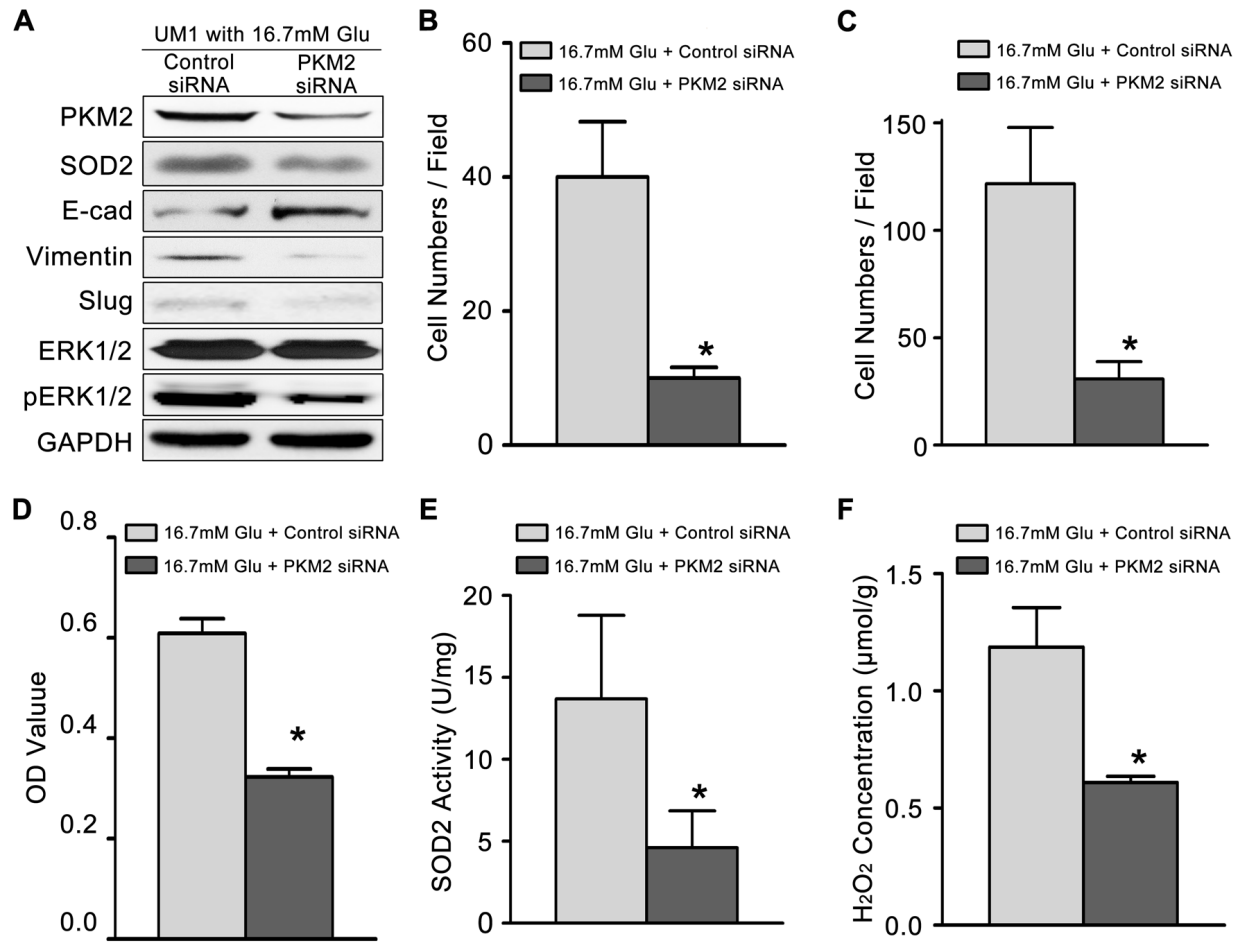
High glucose mediated the migration and invasiveness of TSCC through PKM2 pathway **(A)** The protein levels of PKM2, SOD2, Vimentin, Slug and pERK1/2 were decreased in UM1 cells after knockdown of PKM2 in TSCC cells cultured with 16.7 mM glucose, but the protein level of E-cadherin was decreased. PKM2 knockdown inhibited the migration **(B)** and invasion **(C)** abilities, cell proliferation **(D)**, and the SOD2 activity **(E)** and H_2_O_2_ production **(F)** in UM1 cells cultured with 16.7 mM glucose. ^*^P<0.05 compared with UM1 cells cultured with 16.7mM glucose and transfected with control siRNA.

Then, we knockdown the expression of PKM2 (Figure 3A) in TSCC cells cultured with high glucose (16.7mM). The migration (Figure 3B) and invasion (Figure 3C) abilities, proliferation capacity (Figure 3D), activity of SOD2 (Figure 3E) and production of H_2_O_2_ (Figure 3F) were significantly inhibited in high glucose cultured TSCC cells after knockdown PKM2. The expression levels of metastasis-related proteins (pERK1/2, Slug and Vimentin) and SOD2 were also clearly decreased, while the E-cadherin protein levels were obviously increased after knockdown of PKM2 in TSCC cells cultured with 16.7mM glucose (Figure 3A).

### High glucose promotes tumour growth and lung metastasis *in vivo*

**Figure 4:**
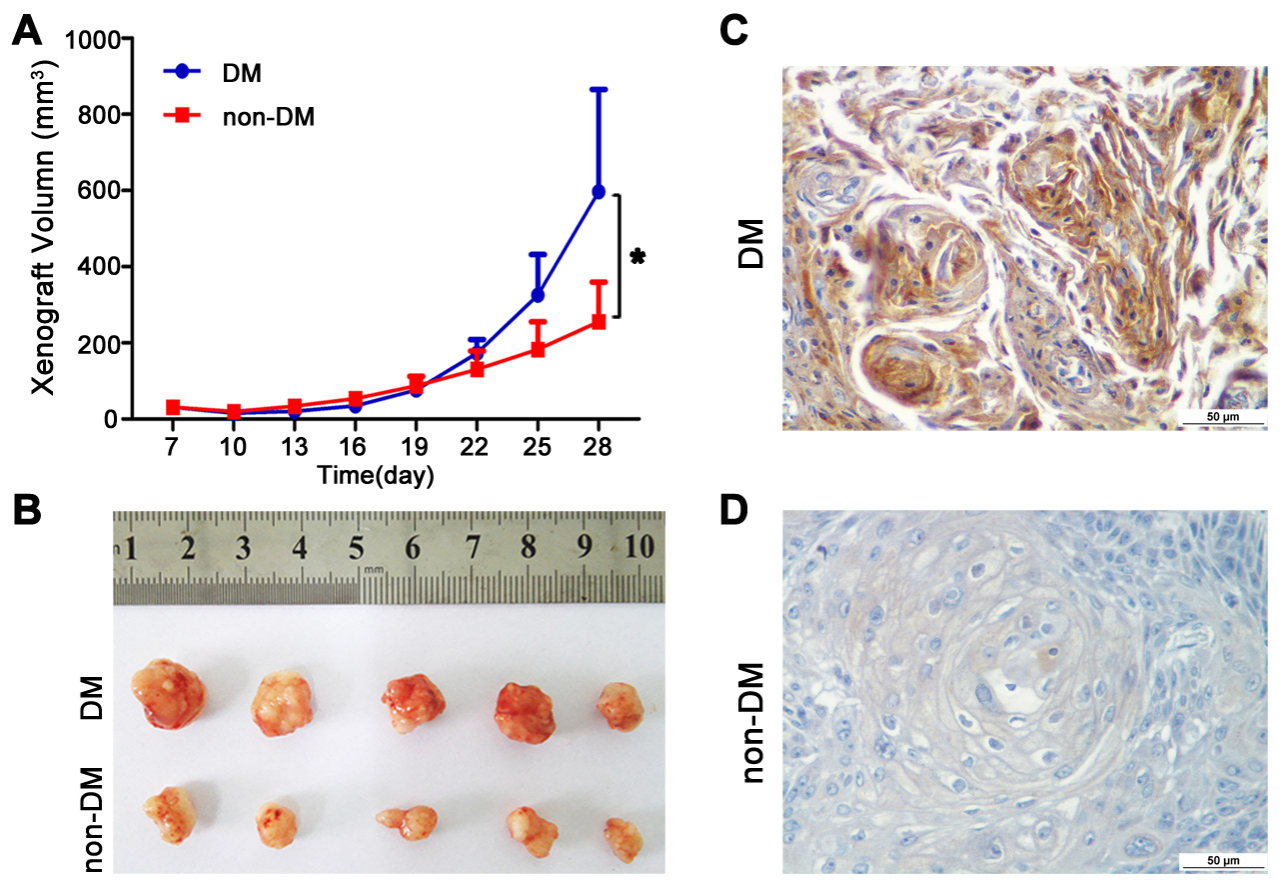
High glucose promotes tumour growth of TSCC *in vivo* **(A-B)** CAL27 cells were inoculated subcutaneously into BALC/C nude mice model of DM. Tumour growth was significantly slower in the non-DM group relative to the DM group. **(C-D)** PKM2 expression was obviously increased in TSCC xenografts from DM group compared with the non-DM group detected by IHC. Scale bar: 50 μm.

**Figure 5:**
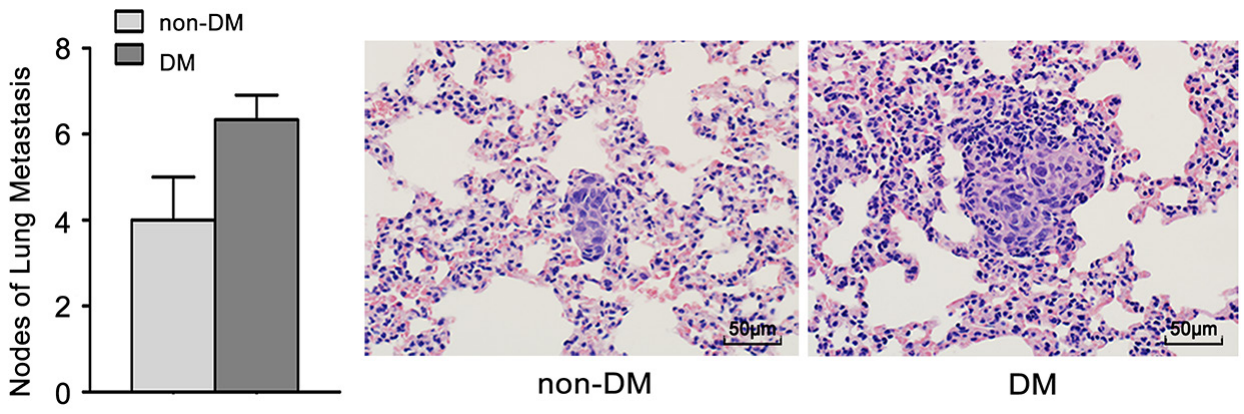
High glucose promotes tumour metastasis of TSCC *in vivo* UM1 cells were injected into the tail veins of DM and non-DM nude mice. The DM mice exhibited a significantly increased number of metastatic nodules relative to the non-DM group. Scale bar: 50 μm.

To further confirm the role of high glucose in the growth and metastasis of TSCC cells *in vivo*, the growth and metastasis of xenograft tumours in nude mice were examined. CAL27 cells were inoculated subcutaneously into nude mice with or without DM (high glucose). As shown in Figure 4A-B, tumour growth was significantly slower in the non-DM group relative to the DM group. The tumour doubling times were 1.3 days (DM group) and 1.9 days (non-DM group), respectively. PKM2 expression was obviously increased in xenografts from DM group (Figure 4C) compared with the non-DM group (Figure 4D). UM1 cells were also injected into the tail veins of DM and non-DM nude mice, and metastatic nodules in the lungs were confirmed histologically and counted. The DM mice exhibited a significantly increased number of metastatic nodules relative to the non-DM group (Figure 5).

## DISCUSSION

More and more increasing evidence indicates a higher cancer risk in individuals with diabetes mellitus [17, 18]. Wang et al. reported that the overall cancer risk in patients with type 2 diabetes mellitus (T2DM) was significantly increased, with SIRs (Standardized Incidence Ratios) of 1.15 and 1.25 in males and females, respectively [18]. He et al [17] also found that individuals with breast cancer and T2DM had more lymph node involvement and that T2DM was associated with poor prognosis in ER/PR-positive or HER2-negative breast cancer. Recently, epidemiological studies also showed that DM may be a risk factor for the development of head and neck squamous cell carcinoma (HNSCC) and may be correlated with a higher incidence of HNSCC [19, 20]. A retrospective cohort study showed that the prevalence rate of DM was higher in patients with OSCC than in the general population in Taiwan (19.1% vs 7.5%). OSCC patients with DM tend to have a lower OS compared with OSCC patients without DM (HR=2.22) [20]. Until now, no reports have investigated the relationship between DM and TSCC. In this study, we found that the prevalence rate of DM in patients with TSCC was 12.84%, which is approximately 1.32 times higher than that in the general population in China, TSCC patients without DM demonstrated a trend of better survival than TSCC patients with DM, as TSCC patients with DM had a 1.38-fold increased risk affecting the 5-year OS compared to patients without DM.

Hyperglycaemia (high glucose), a characteristic feature of diabetes, has been shown to contribute to enhanced cancer risk [10]. Epidemiological evidence suggests that hyperglycaemia in cancer patients contributes to an increased likelihood of tumour recurrence, metastasis or fatal outcome compared to patients with hyperglycaemia [10]. In the present study, we also found that high glucose increases the proliferation, migration and invasion of TSCC cells *in vitro* and promotes tumour growth and lung metastasis *in vivo*.

Given the central role that glycolysis plays in tumour development, elevated glucose levels in the circulation are likely to provide abundant glucose resources and a concentration gradient for convenient usage by cancer cells. Indeed, recent studies have demonstrated that excess glucose induces the expression of glycolytic related gene HK2 and PKM2 [13, 21, 22]. Liu et al [13] found that high glucose stimulated HK2 expression and the migration of wild type (wt) and si-MiaPaCa2 cells in both normoxic and hypoxic conditions. Yang and Lu [22] also reported that high glucose promoted the dephosphorylation of SP1, which increased the DNA binding activity of SP1 and enhanced the expression of PKM2. In our study, a strong correlation was found between DM and the glycolytic enzymes PKM2. PKM2 up-regulation induced by high glucose was also detected *in vitro* by microarray and western blot and in DM animal study. High glucose enhanced the TSCC cell proliferation, migration and invasion and these effect can be abolished after knockdown the expression of PKM2 in TSCC cells. Thus, high glucose promotes cellular metastatic behaviour, which may be related to PKM2.

Previously, many pathways were found to involve hyperglycaemia related to the proliferation and metastasis of cancer cells, such as the TGFβ1/PI3K/AKT signalling pathway [23] and microRNA (miRNA)-associated pathways [24]. In our previous studies, we had found that PKM2 enhances the metastatic potential of TSCC through the SOD2-H_2_O_2_ pathway [16]. SOD2-dependent production of H_2_O_2_ contributed to the migration and invasion abilities of TSCC and to salivary adenoid cystic carcinoma (SACC) via the ERK-Snail (Slug) signalling pathway [25–31]. In the present study, we also found that high glucose induced increased SOD2 expression and activity, intracellular H_2_O_2_ and the expression of pERK1/2 and Slug, whereas, these effect was abolished after knockdown of PKM2 in TSCC cells cultured with high glucose. Thus, These data demonstrate that high glucose promotes the migration/invasiveness of TSCC cells through the PKM2-SOD2-H_2_O_2_ pathway.

## MATERIALS AND METHODS

### Patients and samples

We conducted a retrospective cohort study using data from the First Affiliated Hospital (April 1998-May 2004, cohort #1 and June 2004-September 2014, cohort #2) and data from the Guanghua School of Stomatology (March 2004-September 2014, cohort #3) of Sun Yat-sen University. DM was defined as: (1) fasting plasma glucose levels ≥7.0 mmol/L or (2) 2-h plasma glucose levels ≥11.1 mmol/L. Impaired fasting glucose (IFG) was defined as: (1) fasting plasma glucose levels between 6.1 mmol/L and 7.0 mmol/L or (2) 2-h plasma glucose levels between 7.8 mmol/L and 11.1 mmol/L (Xu et al., 2013). All the patients were diagnosed with TSCC and underwent radical dissection without preoperative chemotherapy or radiotherapy. The clinical characterization of the patients is summarized in Supplementary Table 4. Survival was calculated from diagnosis to the date of the latest follow-up (or death) (2014-09-01). This study was approved by the ethical committee of the first affiliated hospital of Sun Yat-Sen University (2016074).

### Cell culture and transfection

Human TSCC cell lines (UM1, CAL27) were maintained in DMEM supplemented with 10% foetal bovine serum, 1000 U/ml penicillin and 500 mg/ml streptomycin in an incubator at 37°C and 5% CO2. To knockdown PKM2, the cells were seeded in 6-well plates and transfected with PKM2 siRNA or control siRNA (Ribobio, Guangzhou, China) using Lipofectamine™ RNAiMAX transfection reagent (Invitrogen, CA, USA) according to the manufacturer’s instructions. Three sequences of PKM2 siRNA were used, and the sequence that exhibited the best knockdown effect was chosen for further experiments (PKM2 siRNA sequences: 5’-ccauaaucguccucaccaatdt-3’; Control siRNA sequences: 5’-uucuccgaacgugucacgutt-3’). The cells were harvested for functional analysis after 48h transfection.

### *in vitro* cell migration/invasion assays

Transwell assays were performed to assess the cell migration and invasion ability using BD BioCoat Control Cell Culture Inserts and BD BioCoat BD Matrigel™ Invasion Chambers, respectively [26]. Briefly, cells were seeded in the upper Boyden chambers of the cell culture inserts. After 24h of incubation, cells that adhered to the lower membrane were stained with DAPI in the dark, imaged and counted. Three random fields were captured at 200× magnification under a microscope. The number of cells on the lower surface was compared among the groups.

### Cell proliferation assays

Cell proliferation was detected 24h later using a modified Cell Counting Kit-8 (CCK8) assay (Fanbo, Beijing, China) according to the manufacturer’s instructions [32]. Briefly, cells were seeded in 96-well plates at a density of 5×10^3^ cells per well. Then, 10μl of CCK8 solution was added to each well of the plate, which was incubated for 2h in an incubator. The absorbance (optical density, OD) value of each well was determined using a plate reader at a wavelength of 450 nm.

### Western blot analysis

Western blots were performed as described previously [29] using specific antibodies against PKM2, SOD2, E-cadherin (E-cad), Vimentin, members of the Snail family (Slug), extracellular signal-regulated kinase (ERK) 1/2, pERK1/2 and GAPDH (Cell Signaling Technology, MA, USA). GAPDH was used as a control (Cell Signaling Technology).

### SOD2 activity and intracellular H_2_O_2_ concentration

SOD2 activity was determined by a Cu/Zn-SOD and Mn-SOD Assay Kit with WST-8 (Beyotime, China) according to the manufacturer’s instructions [29]. One unit of SOD2 activity was defined as the amount of SOD2 needed to exhibit 50% dismutation of the produced superoxide radical. The final enzyme activity was calculated by normalizing the results to the total protein concentration of the whole protein extract.

The H_2_O_2_ concentration was determined using the PeroXOquant quantitative peroxide assay kit (Pierce, IL, USA) according to the manufacturer’s instructions [29].

### Gene expression profile by microarrayanalysis

UM1 cells cultured with normal glucose (5.6mM) and high glucose (16.7mM) were used to detect the mRNA expression profiles. Total RNA was isolated, labelled, and hybridized to the Affymetrix Human Genome U133 Plus 2.0 GeneChip arrays according to standard, previously reported protocols [33]. The arrays were scanned with a GeneChip Scanner 3000. The scanned array images were processed with GeneChip Operating software (GCOS). The microarray data were pre-processed using Robust Multi-array Analysis (RMA). Experiments were performed in duplicate. The differentially expressed genes were defined as those with a fold change <0.67 (down-regulated) or >1.5 (up-regulated) and a *P* value <0.05. The microarray data has been deposited in Gene Expression Omnibus (GEO) [GEO accession number: GSE99549]. A GO analysis was used to analyse the microarray data.

### Tumourigenesis and metastasis assay in nude mice model of DM

A BALB/C nude mouse model of diabetes was established by a single intraperitoneal injection of STZ [dissolved in 0.1 M sodium citrate buffer (pH 4.5), Sigma-Aldrich, St. Louis, MO, USA] at a dose of 150 mg/kg bodyweight, while control mice received an injection of the same volume of 0.1 M sodium citrate buffer [34]. Three days after the STZ injection, mice with hyperglycaemia (blood glucose levels ≥250 mg/dl) were considered to have DM. For the tumourigenesis, CAL27 cells (5×10^6^/0.2ml) were inoculated subcutaneously into right flanks of 4-wk-old male BALB/c nude mice with or without DM, and the resulting xenografts were measured with a calliper beginning 1 week after inoculation. Tumour volumes were calculated as ½length × width^2^, and the tumour growth curve (y=Ae^kday^) and tumour doubling times (ln2/k) were obtained. The mice were sacrificed after 4 weeks. Immunohistochemistry (IHC) was used to detect PKM2 staining in TSCC xenografts as our previously described [35] using specific antibodies against PKM2 (Cell Signaling Technology, 1:1000). For the metastasis assays, UM1 cells (1×10^6^/0.2 ml) were injected into the tail veins of BALB/c nude mice with or without DM. The animals were sacrificed after 8 weeks, and the metastatic tumours in the lung were assessed as previously described [36].

BALB/C nude mice were purchased from Hunan SJA Laboratory Animal Co. Ltd., Hunan, China. At least 5 mice were used in each group. No mice showed any notable toxic effect or loss of body weight during the experiment. This animal study was approved by the ethics committee of the first affiliated hospital of Sun Yat-Sen University (2016047).

### Statistical analysis

All experiments were performed in triplicate, and the data are presented as the means ± the standard deviation (SD). All statistical analyses were performed using the Statistical Package for the Social Sciences (SPSS, Chicago, IL), Version 19.0. The χ^2^ test was used to analyse the relationship between DM and the clinicopathological characteristics of patients with TSCC. Survival curves were plotted using the Kaplan–Meier method and were compared with the log-rank test. Cox regressions were used for the univariate and multivariate analyses. Correlations between PKM2 expression and DM were tested using Spearman’s rank correlation. The data were analysed with Student’s t test to determine the significance between two groups or with a one-way analysis of variance (ANOVA) to calculate significance among more than two groups. In all cases, *P*<0.05 was considered statistically significant.

## CONCLUSIONS

In this study, we demonstrate that the prevalence rate of DM was significantly higher in TSCC patients than in the general Chinese population, therefore, DM may be a risk factor for the development of TSCC. DM and the expression of PKM2 in TSCC patients were also correlated with each other. High glucose contributed to increased TSCC cells migration/invasion abilities *in vitro* and *in vivo*. High glucose mediated the migration/invasion potential of TSCC through the PKM2-SOD2-H_2_O_2_ pathway. These findings underscore the importance of controlling hyperglycaemia in TSCC patients with DM.

## SUPPLEMENTARY MATERIALS TABLES


