# Supplementary material for: High glucose enhances the metastatic potential of tongue squamous cell carcinoma via the PKM2 pathway
Source: Oncotarget. 2017 Dec 4;8(67):111770–9. doi: 10.18632/oncotarget.22907 (PMC5762358; doi:10.18632/oncotarget.22907)
Supplement: Supplementary file 1 [file oncotarget-08-111770-s001.pdf]

High glucose enhances the metastatic potential of tongue squamous cell carcinoma via the PKM2 pathway

SUPPLEMENTARY MATERIALS

Supplementary Table 1: The relationship between DM and the clinicopathological characteristics of TSCC patients

Characteristics	DM	non-DM	χ^2	*P* value
Gender				
Male	32	254	4.295	0.038
Female	38	177		
Age (years)				
≤40	11	75	0.121	0.728
>40	59	356		
Tumour size				
T_{1+2}	58	343	0.404	0.525
T_{3+4}	12	88		
Lymph node metastasis				
Negative	49	325	0.930	0.335
Positive	21	106		
Clinical stage				
C_{I+II}	43	285	0.588	0.443
C_{III+IV}	27	146		
Histological grade				
Well	36	234	0.199	0.656
Moderate/Poor	34	197		

Supplementary Table 2: Correlations between PKM2 expression and DM were tested using Spearman's rank correlation

	DM	
	r	*P* value
PKM2 expression	0.188	0.025

Supplementary Table 3: Correlations between DM and lymph node metastasis were tested using Spearman's rank correlation

	DM	
	r	*P* value
lymph node metastasis	0.043	0.336

Supplementary Table 4: Clinicopathological characteristics of patients with TSCC

	Cohort #1*	Cohort #2	Cohort #3
	TSCC	TSCC	TSCC
Gender			
Male: n (%)	47(52.81)	89(64.96)	148(53.82)
Female: n (%)	42(47.19)	48(35.04)	127(46.18)
Age			
Median (range)	46(21-77)	57(27-84)	53(24-77)
Tumour size			
T_{1+2}: n (%)	63(70.79)	100(72.99)	232(84.36)
T_{3+4}: n (%)	26(29.21)	37(27.01)	43(15.64)
Lymph node metastasis			
Negative: n (%)	54(60.67)	93(67.88)	216(78.55)
Positive: n (%)	35(39.33)	44(32.12)	59(21.45)
Clinical stage			
C_{I+II}: n (%)	47(52.81)	80(58.39)	192(69.82)
C_{III+IV}: n (%)	42(47.19)	57(41.61)	83(30.18)
Histological grade			
Well: n (%)	52(58.43)	59(43.07)	161(58.55)
Moderate/Poor: n (%)	37(41.57)	78(56.93)	114(41.45)

*Cohort #1 was used in our previous study [28, 35].
